# Oridonin Inhibits Tumor Growth and Metastasis through Anti-Angiogenesis by Blocking the Notch Signaling

**DOI:** 10.1371/journal.pone.0113830

**Published:** 2014-12-08

**Authors:** Yanmin Dong, Tao Zhang, Jingjie Li, Huayun Deng, Yajuan Song, Dong Zhai, Yi Peng, Xiaoling Lu, Mingyao Liu, Yongxiang Zhao, Zhengfang Yi

**Affiliations:** 1 Shanghai Key Laboratory of Regulatory Biology, Institute of Biomedical Sciences and School of Life Sciences, East China Normal University, 500 Dongchuan Road, Shanghai 200241, China; 2 Center for Cancer and Stem Cell Biology, Institute of Biosciences and Technology and Department of Molecular and Cellular Medicine, Texas A&M University Health Science Center, Houston, Texas 77030, United States of America; 3 Biological Targeting Diagnosis and Therapy Research Center, Guangxi Medical University, 22 Shuang Yong Rd, Nanning, Guangxi 530021, China; Medical College of Wisconsin, United States of America

## Abstract

While significant progress has been made in understanding the anti-inflammatory and anti-proliferative effects of the natural diterpenoid component Oridonin on tumor cells, little is known about its effect on tumor angiogenesis or metastasis and on the underlying molecular mechanisms. In this study, Oridonin significantly suppressed human umbilical vascular endothelial cells (HUVECs) proliferation, migration, and apillary-like structure formation *in vitro*. Using aortic ring assay and mouse corneal angiogenesis model, we found that Oridonin inhibited angiogenesis *ex vivo* and *in vivo*. In our animal experiments, Oridonin impeded tumor growth and metastasis. Immunohistochemistry analysis further revealed that the expression of CD31 and vWF protein in xenografts was remarkably decreased by the Oridonin. Furthermore, Oridonin reinforced endothelial cell-cell junction and impaired breast cancer cell transendothelial migration. Mechanistically, Oridonin not only down-regulated Jagged2 expression and Notch1 activity but also decreased the expression of their target genes. In conclusion, our results demonstrated an original role of Oridonin in inhibiting tumor angiogenesis and propose a mechanism. This study also provides new evidence supporting the central role of Notch in tumor angiogenesis and suggests that Oridonin could be a potential drug candidate for angiogenesis related diseases.

## Introduction

Tumor neoangiogenesis not only supplies nutrients and oxygen to enhance tumor growth but also, especially, provides the principal route of tumor metastasis which is the main cause of morbidity and mortality in most cancers [1]. During tumor metastasis, cancer cells escape from the primary tumor, enter into lymphatic or blood circulation (intravasation), and then cross the vessel endothelial cell layer to enter the parenchyma of the target organ. The vasculature endothelial cell layer is a natural barrier for tumor cell trans-endothelial migration and invasion [2]. Therefore, transendothelial migration (TEM) is a critical step in the metastatic dissemination of malignant cells from a primary tumor to distant vital organs [3]. Inhibition of TEM may be an effective strategy of suppressing tumor growth and metastasis.

Among the complex signaling pathways regulating endothelial cell-cell contacts, which determine the permissibility of TEM, the Notch signaling pathway is critical [4]. Notch signaling is an evolutionarily conserved pathway that regulates cell fate decisions during various developmental processes [5]. In mammals, there are five ligands (Jagged 1, 2, Delta-like 1, 3, 4) and four Notch receptors (Notch 1–4), which are expressed on the cell surface [5]. Upon Notch ligand binding to a receptor on an adjacent cell, the intracellular portion of the receptor is cleaved and translocates into the nucleus, leading to the expression of downstream genes such as Hes-1 and HESR1.

A growing body of evidence suggests that Notch is an attractive target to block tumor metastatic progression [6]. Notch mediates communication and interactions between endothelial cells and tumor cells, and promotes tumor angiogenesis [7], [8], [9]. In the tumor microenvironment, Jagged ligands can be induced by tumor-associated growth factors such as VEGF [10], followed by activating Notch expressed in tumor endothelial cells [11]. Curiously, Jagged 2 is the Notch ligand most significantly correlated with overall and metastasis-free survival of breast cancer patients [12]. Vascular endothelial cells express the Notch receptors 1, 2 and 3, and Notch signaling is critical to the proper formation of a functional vasculature [13]. Also, Notch activity was specifically upregulated in the tumor endothelium, suggesting that interfering with Notch activity may negatively affect tumor neoangiogenesis. Several Notch inhibitors such as RO4929097 [14] and MK-0752 [15] have already been used in clinical trials. Therefore, targeting the Notch pathway in endothelial cells might provide a valid strategy for anti-angiogenic therapies [16].

Oridonin, an effective diterpenoid component isolated from the medicinal herb Rabdosia nervosa (Hemsl) [17], has various antibacterial, anti-inflammatory, pro-apoptotic and anti-tumor activities as well as other pharmacological properties [18], [19], [20], [21], [22]. Linda C. Meade-Tollin *et al.* reported Oridonin inhibits formation of capillary-like networks, which implied Oridonin exhibites anti-angiogenesis activity [23]. However, the mechanism of Oridonin action on tumor anigiogenesis remains unknown. In this study, we investigated the mechanism of Oridonin in suppressing tumor growth and metastasis through inhibiting tumor angiogenesis by blocking the Jagged-Notch signaling pathway.

## Material and Methods

### Chemical, Regents and Animals

**Figure 1:**
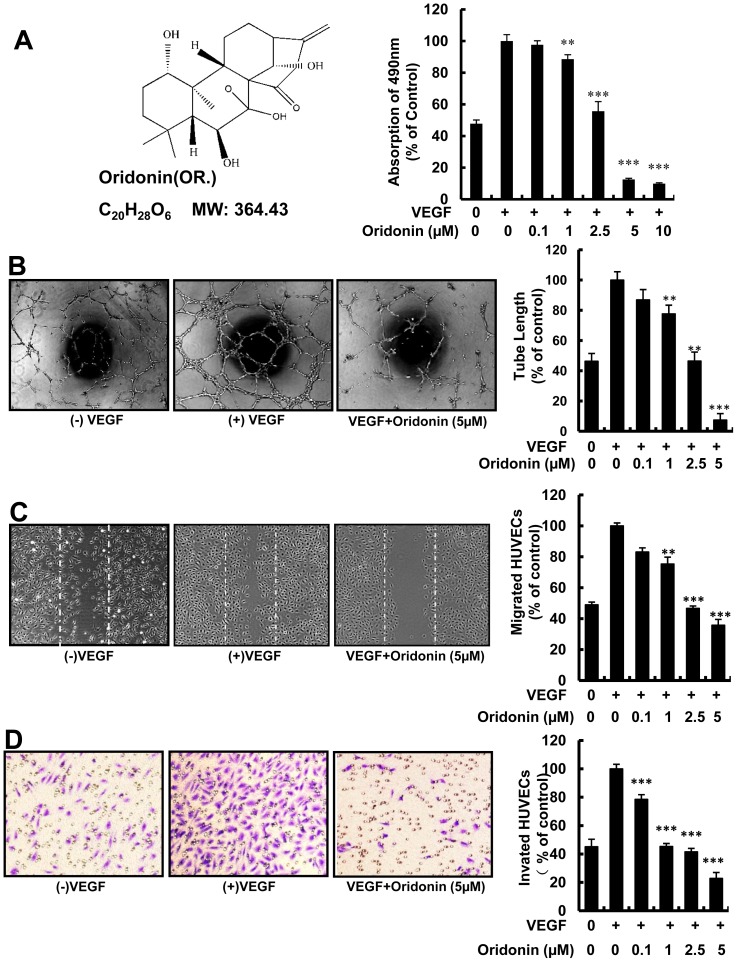
Oridonin inhibited angiogenesis in vitro. (A) The chemical structure of Oridonin (Raddosia rubescens) (left panel) and the MTS assay of HUVECs (right). 5×10^3^ HUVECs were seeded in each well of 96-well plates, and incubated with the indicated concentration of Oridonin after cell adhesion. 490 nm absorbance was measured after 48 hours treatment. (B) Oridonin inhibited VEGF-induced tube formation. 2×10^4^ HUVECs per well were seeded in 96-well plates, and different concentrations of Oridonin were added. Tube like structure length was measured after incubating for 8–10 hours. (C) Oridonin significantly inhibited VEGF-induced HUVECs wound healing. The cells pretreated with mitomycin C to inhibit proliferation before inducing migration. Dotted lines indicated the scraped area. Decreased migration was significant at 1 µM,and the difference is highly significant between control and 5 µM of Oridonin. (D) Oridonin significantly inhibited VEGF-induced Modified Boyden chamber migration. Arrows pointed to the migrated cells. 4×10^4^ HUVECs were seeded in the upper chamber, and after 4 hours migrated cells were stained with crystal violet after fixation with Paraformaldehyde. (*, P<0.05; **, P<0.01; ***, P<0.001).

Oridonin (Fig. 1A left panel) (purity more than 98%) was purchased from Shanghai Zhanshu Chemical Technology Co. Ltd in China. VEGF was obtained from R&D Systems provided by Biological Resources Branch, NCI-Frederick Cancer Research and Development Center. Matrigel was purchased from BD Biosciences (San Jose, CA). Notch inhibitor DAPT was purchased from Sigma (Sigma-Aldrich, Inc., St Louis, Mo, USA).

Sprague Dawley (SD) rats, C57BL/6, BALB/c and nude mice were purchased from National Rodent Laboratory Animal Resources, Shanghai Branch of China. Mice were maintained according to the NIH standards established in the Guidelines for the Care and Use of Experimental Animals, and all of the experimental protocols were approved by the Animal Investigation Committee of the Institute of Biomedical Sciences and School of Life Sciences, East China Normal University.

### Cell Culture and Proliferation Assay

HUVECs (ScienCell Research Laboratories, San Diego, CA, USA) were purchased from Science Research Laboratories and cultured in complete ECM (Sciencell) supplemented with 5% FBS. HCT116 cells were obtained from the American Type Tissue Collection (ATCC, Manassas, VA, USA) and maintained in DMEM supplemented with 10% FBS (Gibco BRL Life Technologies, Eggenstein, Germany). 4T1 mammary carcinoma cell was purchased from ATCC and maintained in RPMI-1640 medium supplemented with 10% FBS, 1% Glutamax-1 and 1% penicillin-streptomycin. All cells were maintained at log phase at 37°C with 5% carbon dioxide. Cell proliferation was determined by the Promega CellTiter 96 (Promega, Madison, WI, USA) nonradioactive cell proliferation assay according to manufacturer's instruction [24]. All experiments were performed in triplicate and repeated at least three times.

### Tube Formation and Migration Assay

*In vitro* angiogenesis was assessed with tube formation and migration assays. Briefly, 1×10^4^ HUVECs were seeded on Matrigel with or without different concentrations of Oridonin followed by the addition of VEGF (20 ng/ml). After about 8 hours, photomicrographs were taken with an OLYMPUS inverted microscope. Tubular structures were quantified by Image-Pro Plus 6.0 software, and the inhibition percentage was expressed using untreated wells as 100%. HUVEC migration was determined with a wound healing migration assay and a modified Boyden chamber assay. Confluent HUVECs were pretreated with Mitomycin C for 2 hours before incubating with VEGF (20 ng/ml) and Oridonin for about 8–12 hours. Migrated cells were photomicrographed and counted manually. The modified Boyden chamber model (Transwell, 8.0 µm pore size; Costar) was used as previously described [25]. HUVECs at 80% confluence were serum-starved for 24 hours. Next, HUVECs (4×10^4^) in 100 µl of serum-free medium were plated in the transwell insert. Then 600 µl of fresh basic ECM medium containing 10 ng/ml vascular endothelial growth factor (VEGF) and different concentrations of Oridonin were added into the bottom well. After 4 hours of incubation, cells were washed with PBS to remove the un-invaded cells, fixed with 4% paraformaldehyde and stained with crystal violet. Migrated cells were imaged using Olympus IX70 inverted microscope connected to a DXM1200 digital camera and counted manually.

### Aortic Ring Spreading Assay

Aortic ring spreading assay was performed as previously described with modification [26]. 48-well plates were coated with 100 µl of Matrigel per well and polymerized at 37°C for 30 minutes. Aortic rings harvested from 6 to 8-week-old Sprague-Dawley rats were plated in the wells and overlaid with 100 µl of Matrigel for sealing. VEGF (100 ng/ml) in serum-free culture medium, with or without 5 µM Oridonin was added. The medium was changed every two days. Microvessel-like properties of sprouting structures were observed and photographed on the 7^th^ day and counted by Image-Pro Plus 6.0 software.

### Mouse Corneal Micropocket Assay

Corneal angiogenesis was assessed as described previously [27]. Briefly, in one eye of 6 to 8-week-old male C57BL/6 mice, corneal micropockets were created using a modified needle. Micropellet containing 250 ng VEGF was implanted into each corneal pocket. After injecting 5 mg/kg Oridonin intraperitoneally for seven days, eyes were imaged under the microscope. The length and clock number of new blood vessels were surveyed, and the area of neovascularization was calculated using the formula Area (mm^2^)  = 0.2×3.14×VL×CN, where VL is the maximal vessel length extending from the limbal vasculature toward the pellet, CN is the clock hours of neovascularization and 1 clock hour equals 30 degrees of arc.

### Xenograft Mouse Tumor Model

Male nude mice, 5–6 weeks old, were inoculated subcutaneously in the right flank with 5×10^6^ HCT116 cells suspended in 50 µl PBS. When tumor volume reached ∼150 mm^3^, mice were randomly assigned to the Oridonin treatment group (n = 8) or the control (DMSO) group (n = 8). Oridonin (7.5 mg/kg) was administered daily by intraperitoneal injection. Tumor volume was determined using digital vernier caliper measurements and the formula: A×B^2^×0.52, where A is the longest diameter of the tumor and B is the shortest diameter of the tumor.

### Spontaneous Metastasis Model and Hematoxylin & Eosin Staining

The metastasis model was as previously reported [28], orthotopic injections were performed under anesthesia by using sodium pentobarbital. An incision was made through the abdominal muscles between 4^th^ and 5^th^ papilla to expose the mammary fat pat (MFP). 4T1 cells (1×10^5^) in 50 µl PBS were injected into the MFP. Based on the primary tumor size, mice were segregated into groups for the appropriate treatments. On the day 7, Oridonin was injected intraperitoneally every two days. Lungs were dissected and fixed in 10% formaldehyde when the mice were agonal. Paraffin-embedded lungs were cut for H & E staining. Slides were examined under a light microscopy and pictures were taken at 200× magnification.

### Immunohistochemistry

Paraffin-embedded sections of tumor tissues and lungs were used for immunohistochemistry staining performed as previously described [29], [30]. Immunohistochemistry analysis was performed on 5-µm sections of formalin-fixed paraffin-embedded colon tumors derived from mice injected with HCT116 cancer cells. Then 50 serial sections were cut from each tissue, and every 10th tumor section was stained for expression of the endothelial cell-specific marker CD31 and Von Willebrand Factor (vWF) by using an anti-CD31 (Lifespan Biosciences, Inc., Seattle, WA, USA) and anti-vWF polyclonal antibody (Millipore Corporation, Temecula, California, USA). Lung sections were stained with anti-CD31 and anti-Pericentrin (Abcam plc. Temecula, California, USA) polyclonal antibodies. Images were recorded under the Leica DM 4000B photomicroscope and integrated optical density (mean IOD) of blood vessels in tumor sections was analyzed using Image-Pro Plus 6.0 software. Angiogenetic vessels consisting of at least two visible cells in a crosssectional view were scored as positive.

### Immunofluorescence

After treating with Oridonin, HUVECs grown on gelatin-coated glass coverslips were fixed in 4% (w/v) paraformaldehyde and permeabilized with 0.1% Triton-X 100 in PBS. F-actin was visualized by sequential incubation with indicated antibody overnight at 4°C (Jackson Immunoresearch Laboratories, West Grove, PA, U.S.A.). Pictures were acquired using a confocal microscope (Leica Instruments, IN, U.S.A.) controlled by the LAS AF advanced 1.9.0 software (Diagnostic Instruments).

### Transendothelial Migration of Tumor Cells

Tumor cell transendothelial migration was performed as previously described [2]. Approximately 2×10^5^ HUVECs were added to matrigel-coated 24-well Transwell inserts and grown to confluence for 48 hours, with daily replacement of fresh culture medium. Then, 1×10^5^ 4T1-GFP cells with different concentration of Oridonin were added onto the HUVEC monolayer and incubated for 18–20 hours. Then the non-migrated cells were removed using a cotton swab. To assay migration, the migrated cells were photographed and counted manually.

### Real-time Quantitative Polymerase Chain Reaction

HUVECs were starved for 12 hours and exposed to various concentrations of Oridonin for 12 hours and then induced with VEGF (100 ng/ml) for 10 minutes. Total RNA was isolated from treated HUVECs with Trizol (Invintrogen). 1 µg of total RNA was high performance reverse transcribed into cDNA in a volume of 20 µl with reverse transcriptase and oligo dT primers (Promega, Madison, USA) according to the manufacturer's manual. The cDNA of β-actin served as the endogenous control. A quantitative fluorogenic SYBR Green (Takara, Otsu, Shiga, Japan) used for real-time quantification in an ABI prism 7000 sequence detection system. Primer sequences for *Jagged 1*, *Jagged 2*, *Dll-1*, *Notch1*, *Hes-1* and *HESR1* are summarized in the Table 1 [31]. PCR cycles were 5 minutes at 95°C, followed by 40 cycles with an annealing temperature of 56°C.

**Table 1:** Sequences of primers used in Q-PCR reactions.

Genes	Forward (5'-3')	Reverse (5'-3')
*Jagged 1*	CAACACGGTCCCCATCAAG	TACTTCAGAATTGTGTGTCCTTATTTTAGA
*Jagged 2*	GGCACTCGCTGTATGAAAGGA	GCACAACCTCTGGTAACAAACG
*Dll-1*	GGTCATGGAGTTGTCATTCGTCTA	TATCATTTCCTGTGCCAACTCTTTT
*Notch 1*	CCGCAGTTGTGCTCCTGAA	ACCTTGGCGGTCTCGTAGCT
*Hes-1*	AGCGGGCGCAGATGAC	CGTTCATGCACTCGCTGAA
*HESR 1*	CTTGAGTTCGGCTCTGTGTTCC	GATGCCTCTCCGTCTTTTCCT

### Immunoblot Assay

HUVECs were collected and incubated in lysis buffer (50 mM Tris-HCl, pH 7.4, 150 mM NaCl, 1% Triton X-100, 0.1% sodium dodecyl sulfate (SDS), 1 mM ethylenediaminetetraacetic acid (EDTA), supplemented with protease inhibitors (10 mg/ml leupeptin, 10 mg/ml aprotinin, 10 mg/ml pepstatin A, and 1 mM of 4-(2-aminoethyl) benzenesulfonyl fluoride) and phosphatase inhibitors (1 mM NaF and 1 mM Na_3_VO_4_) for 30 minutes on ice. Lysates were centrifuged at 12,000 rpm for 20 minutes at 4°C. Lysates containing 40 µg of protein were fractionated by SDS-polyacrylamide gel electrophoresis (PAGE) and electrotransferred to a PVDF membrane. The blocked membranes were then immunoblotted with primary antibodies (1∶1000 dilution) of VE-cadherin, Jagged 2, Notch 1, cleaved Notch 1 (Cell Signaling Tech., Beverly, MA, U.S.A.), Hes-1 (Millipore., Single Oak Drive, Temecula, CA, USA), HESR 1, and β- actin (Sigma, St. Louis, MO, U.S.A.). The proteins were visualized using enhanced chemiluminescence by secondary antibody.

### Statistical Analysis

Statistical analysis was performed with Microsoft Excel software. The values *in vitro* experiments were expressed as means ±SE. The Student t-test for independent analysis was applied to evaluate the difference between treatment and control groups. All experiments were repeated at least three times except animal experiment. A value of p<0.05 was considered statistically significant.

## Results

### Oridonin Inhibited Endothelia Cell Proliferation

To detect the effect of Oridonin on angiogenesis *in vitro*, we first examined the potential anti-proliferation activity of Oridonin on HUVECs with MTS assay. Result showed that Oridonin significantly inhibits VEGF induced HUVEC proliferation in a dose-dependent manner, with the IC50 at about 2.5 µM (Fig. 1A right panel).

### Oridonin Inhibited Tube Formation and Migration of HUVECs in vitro

As tube formation is a critical step in angiogenesis, we investigated whether Oridonin affected HUVEC angiogenic activity *in vitro* with tube formation assay. We found that tubular structures on Matrigel decreased over 90% after treating with Oridonin (Fig. 1B). Then we examined the effects of Oridonin on migration of HUVECs *in vitro*. When incubated with VEGF and Oridonin, HUVEC wound healing and transwell migration were suppressed in a dose-dependent manner with the IC50 at 2.5 µM (Fig. 1C and D). Therefore, Oridonin inhibits endothelial cell proliferation, migration, and tube formation, three activities essential for angiogenesis.

### Oridonin Suppressed Microvessel Formation in the Rat Aortic Ring ex vivo

**Figure 2:**
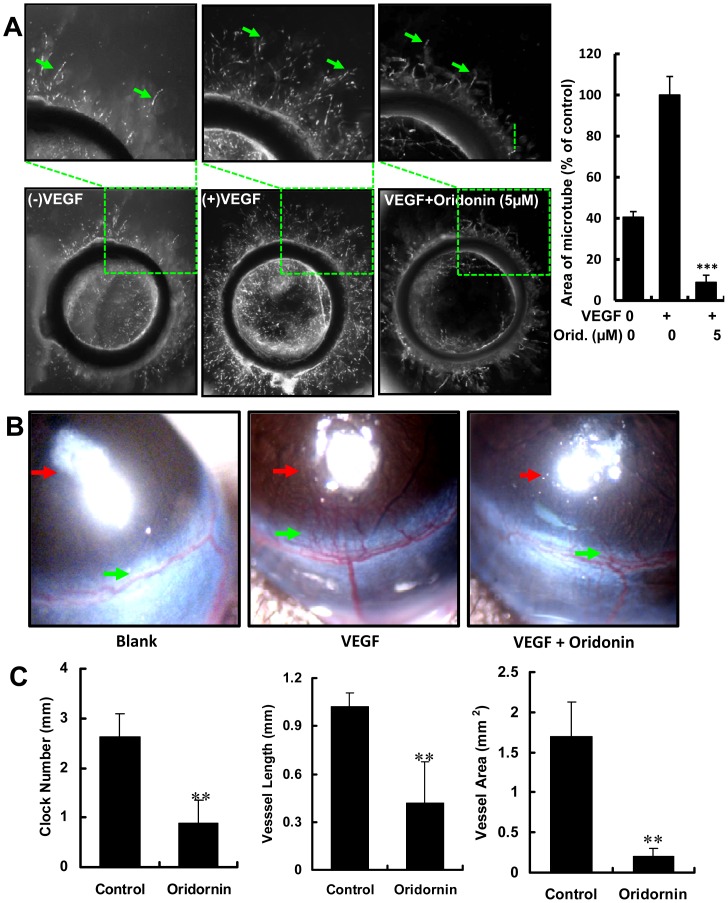
Antiangiogenic activity of Oridonin *ex vivo* and *in vivo*. (A) Oridonin inhibited VEGF-induced micro-vessels formation. The right panel shows quantitation of micro-tube area. (B) The effects of Oridonin in the VEGF-induced mouse corneal vascularization assay. Red arrows indicate the micropellets that carry VEGF, and the green arrows indicate the neoangiogenic micro-vessels. (C) Quantitation of mouse corneal assay. The Clock number of the angiogenesis area is on left panel, vessel length of the neoangiogenic vessels (middle panel), and area of angiogenesis vessels (right panel). Here, one clock number represents a 30 degree arcs, and the area is the product of vessel length and clock number. (**, P<0.01; ***, P<0.001).

In the rat artic ring assay, the effect of Oridonin on the sprouting of vessels from aortic rings upon VEGF (20 ng/mL) stimulation was examined. After incubation with 0.1% DMSO and Oridonin for seven days, there were significantly fewer neo-microvessels from aortic rings in the Oridonin-treated group than those in control group. 5 µM Oridonin effectively block VEGF-induced microvessel sprouting, with a reduction of more than 90% (Fig. 2A).

### Oridonin Inhibited VEGF-induced Angiogenesis in vivo in the Mouse Corneal Neovascularization Model

To further investigate whether Oridonin inhibited VEGF-induced angiogenesis *in vivo*, Oridonin was tested in the mouse cornea model. We found that Oridonin markedly inhibited VEGF-induced neovascularization (Fig. 2B). Quantification analysis revealed the significant anti-neovascularization effect of Oridonin, with an 88% reduction of vascularization area (N = 8, P<0.01) (Fig. 2C right panel). At the same time, clock number decreased by 66.7% and vessel length by 60% upon treatment with Oridonin (Fig. 2C left panel and middle panel). These findings demonstrated that Oridonin was a potent inhibitor of angiogenesis *in vivo*.

### Oridonin Suppressed Tumor Growth and Angiogenesis in Solid Tumor

**Figure 3:**
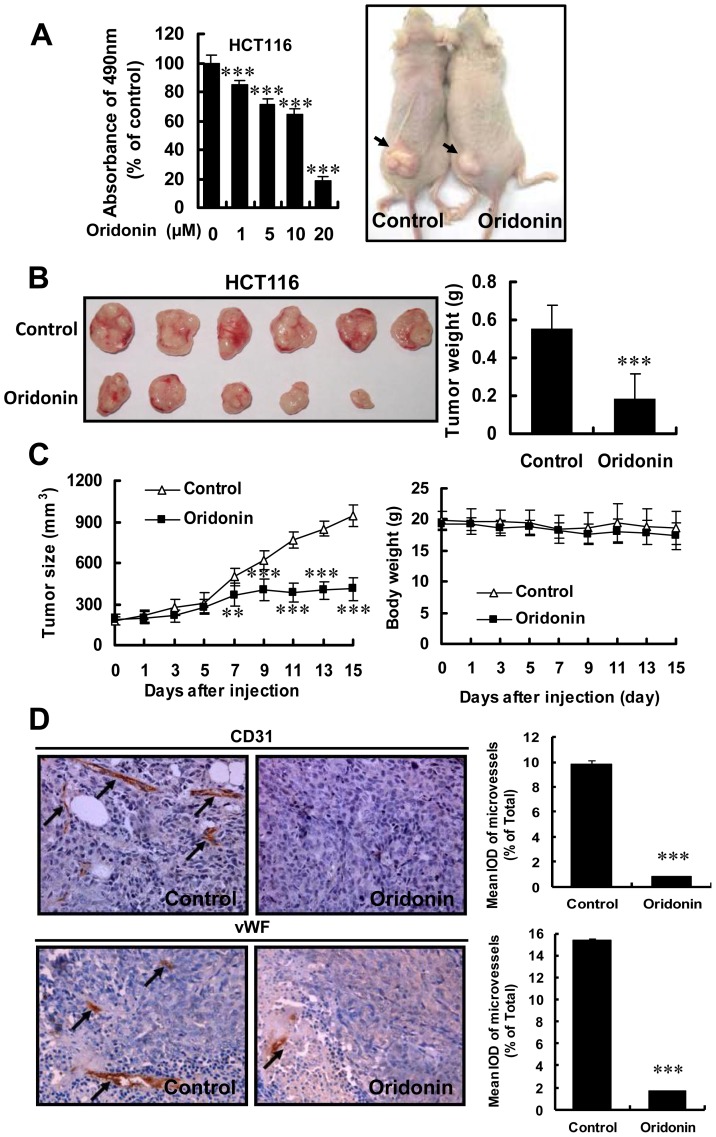
Oridonin suppressed tumor growth through antiangiogenic activity. (A) The MTS proliferation assay of HCT116 (left panel) and the representative mice with tumor after treating with DMSO or Oridonin (right panel). (B) Photographs of DMSO and Oridonin treated group tumors, along with the graph of tumor weight. (C) The mean tumor size and the body weight of control and Oridonin groups. (D) Immunohistochemistry of tumor slides stained with antibodies against vWF and CD31; arrows showed new blood vessels in the tumor, with the statistical results of microvessels on the right. Mean integrated optical density (mean IOD) of blood vessels accords to the following formula: mean IOD = IOD/area of the tumor section. (**, P<0.01; ***, P<0.001).

Our results above show significant effects of Oridonin on *in vitro* endothelial cell parameters relevant to angiogenesis (Figs. 1 and 2) as well as *in vivo* inhibition of corneal vascularization (Fig. 2B and C). However, the tumor environment is complex and characterized by down-regulation of multiple signal transduction pathways that may impede the efficacy of compounds that function in more normal settings. Therefore, to investigate the value of Oridonin as an anti-angiogenic therapeutic compound in tumors, we evaluated the ability of Oridonin to block tumor growth and angiogenesis in the subcutaneously implanted HCT116 colorectal carcinoma tumor model [32]. When tumors became palpable, mice were treated with either Oridonin (7.5 mg/kg per day) or DMSO for 15 days. We found that tumor size (Fig. 3A, right panel and Fig. 3B-3C, left panels) was significantly reduced in the Oridonin treated group. The mean tumor weight of the Oridonin – treated group was also much smaller that of the control group (Fig. 3B right panel). At the same time, there was no significant difference between control and Oridonin-treated mouse body weight (Fig. 3C, right panel), implying the potential for few side-effects of Oridonin at the therapeutic dosage. Tumor sections stained with anti-CD31 and anti-vWF antibodies revealed that Oridonin inhibited new blood vessels as well as pruned preexisting tumor vessels (Fig. 3D). To test the effect of Oridonin on HCT116 tumor cells, additional analysis with MTS proliferation assay was performed, and results showed that Oridonin inhibited HCT116 tumor cells proliferayion. However the IC50 of Oridonin on HCT116 was 10–20 µM (Fig. 3A left panel), which was more than that on HUVECs (IC50 <5 µM) (Fig. 1A right panel), suggesting that Oridonin was more effective in inhibiting proliferation in HUVECs than in HCT116 cells at the same concentration. These results suggested that Oridonin reduced tumor growth mainly via inhibiting tumor angiogenesis.

### Oridonin Block Tumor Cell Extravasation across the Endothelium

**Figure 4:**
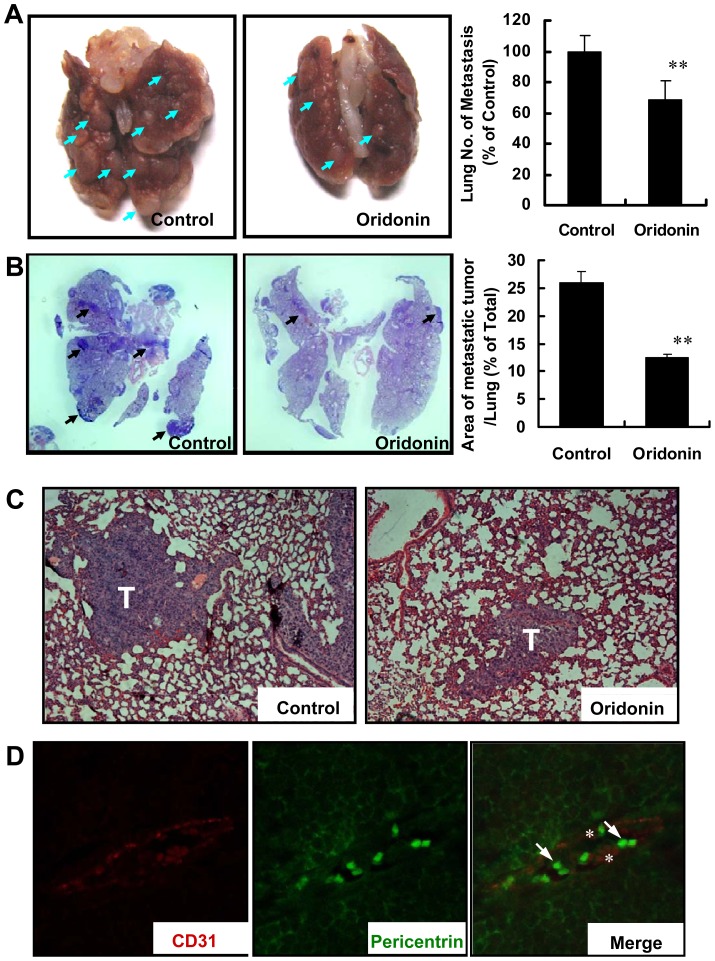
Oridonin inhibited tumor metastasis through blocking tumor cell trans-endothelium. (A) Right panel, representative lungs of mice (n = 6 per group) from the metastasis model. Arrows indicate the metastasis nodes. Quantitation is shown in the right panel. (B) The photographs of haematoxylin and eosin (H&E) staining of control (DMSO) or treatment (Oridonin) group lung tissues are shown. The graph shows a comparison of metastatic area in control and Oridonin treated group (n = 6). (C) The magnified images of H&E staining of lungs in Fig. 4B. “T” indicates the metastatic tumor nodes in the lung. (D) Typical images of lungs in Oridonin-treated breast cancer metastasis. As showed by immunofluorescence double staining using anti-CD31 (red) and anti-pericentrin (green) antibodies, all breast cancer cells are inside of blood vessels. The asterisk shows red blood endothelial cells. (**, P<0.01).

It is well known that malignant tumors depend on neovascularization for their metastasis [33]. To test whether Oridonin affects tumor metastasis, we performed tumor metastasis assays, injecting 4×10^5^ 4T1 breast cancer cells into the mammary fat pad of BALB/c mice. In all injected BALB/c mice, the primary tumor burden was high, with no significant difference between treated and control mouse tumor size, 754 mm^3^ and 793 mm^3^, respectively at the 23^rd^ day (data not shown). To our surprise, metastatic node formation was inhibited in the Oridonin group (51 nodes in average) compared to the control group (72 nodes in average); a representative lung from each experimental group was shown in Fig. 4A. In additional analysis, normal tissue and metastatic nodules in the lung were obvious after H &E staining, as being sparse or compact, respectively (Fig. 4B). Metastases comprised 26% of lung area in control mice, but was significantly reduced in the Oridonin group (12%, n = 6, p<0.01). Typical areas of H&E stained lungs in control and Oridonin-treated groups are shown in Fig. 4C. Individual tumor cells in the Oridonin-treated mice were found inside the blood vessels (data not shown). To confirm that these cells were tumor cells, we performed immunofluorescence staining for pericentrin, a centrosomal protein used to detect neoplastic cells [34]. Breast tumor cells in control lungs with invasive metastases show a high level of centrosomal abnormalities by immunofluorescence staining for pericentrin (S1 Figure and Fig. 4D). In Oridonin–treated mice, tumor cells (green) with centrosomal abnormalities were only located inside CD31-stained blood vessels (red) (Fig. 4D). No tumor cells were found outside the vessels. These results may imply that Oridonin inhibits metastasis formation *in vivo* by inhibiting the migration/invasion of tumor cells through the endothelium.

### Oridonin Regulated HUVEC Cell-cell contacts and Inhibited Tumor Cell Transendothelial Migration In Vitro

**Figure 5:**
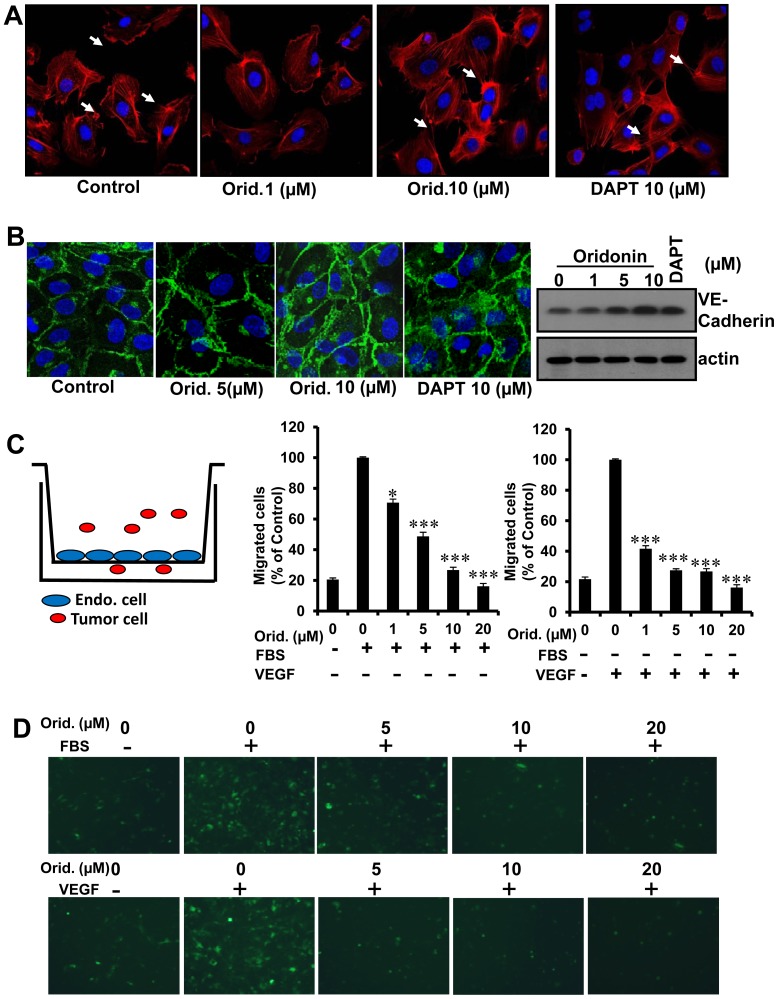
Oridonin increased the cell-cell connections of HUVECs and decreased tumor cell transendothelial invason. (A) HUVEC morphology and cell-cell contacts by immunofluorescence assay. Arrows show the contact of endothelial cell edge with different concentrations of Oridonin. (B) HUVECs were treated with Oridonin or DAPT for 24 hours. Cells were fixed and stained with VE-cadherin. Photographs were obtained through a confocal microscope (left panel). HUVECs were treated with Oridonin or DAPT for 24 hours, and cells were harvested. VE-cadherin expression was examined by western blot analysis (right panel). (C) Transendothelial migration of 4T1 breast tumor cells with FBS or VEGF stimulation of HUVECs. 2×10^5^ HUVECs were grown to confluency for 48 hours on the Transwell membrane. HUVECs were treated with FBS or VEGF (20 ng/ml) for 18 hours. 1×10^5^ 4T1-GFP cells serum-starved overnight were added on the monolayer of HUVECs and incubated for 6 hours. After migrated cells were fixed and stained, the photographs were acquired. (D) The represent photographs of Fig. 5C. (*, P<0.05; **, P<0.01; ***, P<0.001).

Cell-cell contact is indispensable during cell motility [35] and actin fibers underlie early cell-cell contacts [36]. To investigate the mechanism of Oridonin inhibition of EC migration and tumor cell TEM, we first performed F-Actin and detected the expression of VE-cadherin. VE-cadherin is crucial for the maintenance and control of endothelial cell-cell contacts [37]. Results showed that the morphology and cell junction of HUVECs were changed after treating with Oridonin. There are more cell-cell connections with Oridonin (Fig. 5A and B). Previous studies showed that Notch signal pathway contributes to cell-cell contact [38], [39]. Therefore, we treated HUVECs with the Notch inhibitor DAPT and found that the effects of HUVEC treatment with 10 µM DAPT was similar to that of Oridonin (Fig. 5A and B). These results suggested that the molecular mechanism of Oridonin biological effects in HUVECs may involve Notch pathway signaling.

Tumor cell transendothelial migration is a crucial step in both the intravasation of cells from primary tumors into the vasculature and in extravasation of circulating tumor cells into suitable secondary sites leading to tumor metastatic colonization [40]. To further study whether Oridonin affects TEM, we measured the transendothelial migration of 4T1 breast cancer cells. In order to exclude the confounding effects of differing HUVEC densities, the monolayer in each chamber was confirmed to be confluent by visual inspection for each experiment. As showed in Fig. 5C and D, 4T1 tumor cell transendothelial migration could be enhanced by FBS or VEGF and Oridonin decreased VEGF-induced migration more effectively than FBS-induced trans-HUVEC migration, with an IC50 of less than 1 µM. These data showed that Oridonin inhibits an important step in breast cancer cell transendothelial migration *in vivo*.

### Oridonin Blocked the Jagged-Notch Signaling Pathway in Endothelial Cells

**Figure 6:**
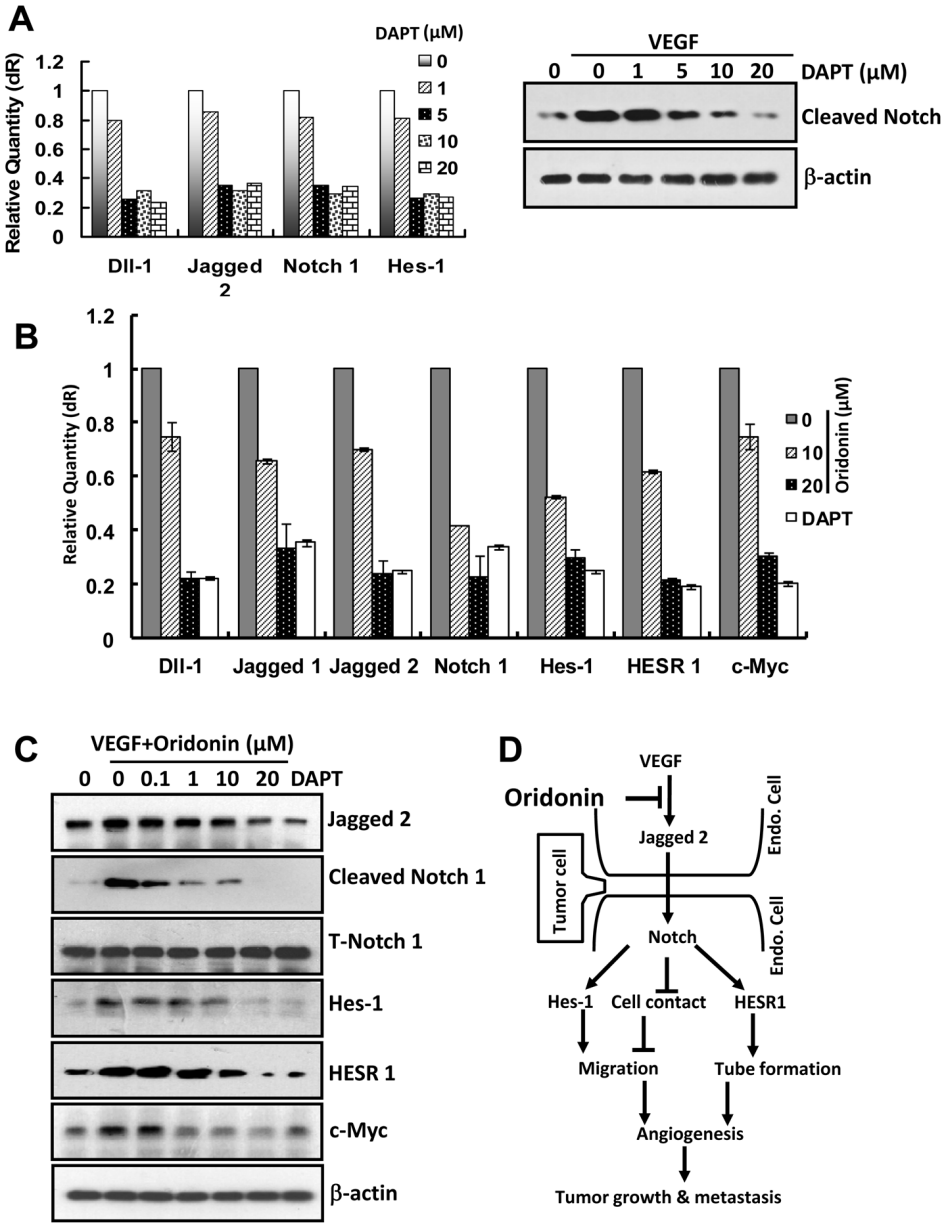
Notch pathway was regulated in Oridonin in HUVECs. (A) DAPT blocked Notch gene transcription and Notch activity. (B) and (C) Oridonin remarkably inhibited expression of key ligands, receptors and downstream genes (Fig. 6B, Q-PCR) and proteins (Fig. 6C, Western blot) involved in the Jagged-Notch Signaling Pathway. (D) Schematic diagram of Oridonin inhibiting tumor growth and tumor metastasis by regulating the Jagged-Notch signaling pathway in HUVECs.

One published signaling cascade mediating VEGF signaling is the Notch pathway. We were interested to determine whether Oridonin could affect Notch signaling in endothelial cells. First, we verified inhibition of Notch signaling in HUVECs using a known Notch inhibitor, DAPT. To examine the effects and concentration- dependence of DAPT on Notch signaling in HUVECs, we performed Q-PCR and western blot assays. 20 µM DAPT suppressed the mRNA expression of Dll-1, Jagged2, Notch1 and Hes-1, which are involved in the Notch pathway (Fig. 6A left panel). At the same time, DAPT inhibited VEGF-induced Notch activity (Fig. 6A right panel). Thereafter we used 20 µM DAPT as the positive control in the gsubsequent assays to measure Oridonin effects on Notch signaling. As expected, Oridonin remarkably inhibited key ligands, receptor and downstream genes and protein expression involved in Jagged-Notch Signaling Pathway (Fig. 6B and C). In particular, note the inhibition of both Jagged2 ligand and VEGF-stimulated Notch activation at 10 µM Oridonin, suggesting potency of VEGF/Notch inhibition by Oridonin at concentrations relevant to effects on endothelial cell functions *in vitro*. Therefore, Oridonin inhibition of angiogenesis may occur through blockage of VEGF stimulation of Jagged2 and Notch activation.

## Discussion

Angiogenesis is required for tumor growth and metastasis [25]. Identification of novel angiogenesis inhibitor will benefit drug discovery for treating angiogenic diseases, such as tumor. Here, we found that Oridonin not only inhibited angiogenesis in the mouse cornea assay, the golden standard for angiogenesis, but also suppressed rat artery ring sprouting. In addition, Oridonin inhibited tube formation which was consistent with a previous result that Oridonin could inhibit capillary-like networks formation of human dermal microvascular endothelial cells [23].

Over 90% of deaths from solid tumors are attributable to tumor metastasis [41]. Cancer metastasis is a multistep process involving many types of cell-cell interactions. In the tumor metastatic cascade, tumor cells initially attach to vascular endothelium and intravasate, followed by circulation and extravasation from the blood stream. To form metastases, migrating tumor cells must overcome the endothelial barrier [42] which is accompanied by drastic changes in cytoskeletal organization in neighboring endothelial cells [43]. Therefore, transendothelial migration of tumor cells is a crucial step of metastasis and inhibitors of transendothelial migration may be an attractive way of blocking metastasis. Here, we found that Oridonin promoted HUVECs cell-cell connections (Fig. 5A), which may have prevented tumor cells from crossing the endothelial cell layer. At the same time, data presented here provided evidence that Oridonin more significantly inhibited VEGF-induced transendothelial migration than that of FBS-induced in breast cancer cell (Fig. 5B), implying that Oridonin has potential specificity for a downstream target of VEGF signaling. These results, together with the effects of tumor metastasis and growth inhibition, suggested that inhibition of transendothelial migration of tumor cells is an important aspect of blocking breast tumor metastasis by Oridonin.

Among the molecular mechanisms involved in tumor angiogenesis and metastasis, accumulating evidence suggests that Notch is a key regulator of tumor angiogenesis and metastasis [6], [44], [45]. Disruption of Notch has been implicated in multiple tumor types [46]. In breast cancer, evidence from *in vitro* experiments, mouse models and human tumor samples indicated that Notch plays a predominantly oncogenic role [46], suggesting the importance of Notch signaling pathways during tumor development. VEGF and Notch are interacting signaling pathways in tumor angiogenesis [47]. It is well established that the VEGF acts as a potent activating stimulus for angiogenesis, whereas Notch appears to help guide cell fate decisions that appropriately shape this activation. These reports suggest that VEGF and Notch may be valid therapeutic targets in cancer [48]. In the present study, we demonstrated that Oridonin inhibited the expression of Jagged2, Notch, and their downstream genes (Fig. 6B), similar to the effect of the Notch inhibitor DAPT (Fig. 6A). Furthermore, we found that Oridonin down-regulated the activity of Notch (Fig. 6C). These results suggested that the above biological functions of Oridonin in inhibiting tumor angiogenesis and metastasis was accomplished through blocking the Jagged-Notch pathway.

In conclusion, our results indicate that Oridonin inhibited tumor growth and metastasis via suppressing tumor angiogenesis by blocking the VEGF induced Jagged-Notch signal pathway. Oridonin may be an effective anti-tumor candidate therapeutic by regulating Jagged-Notch activity in cancer therapy.

## Supporting Information

S1 Figure **Oridonin inhibited tumor metastasis through blocking tumor cell trans-endothelium.** (A) Typical images of lungs in control group. No breast cancer cells are inside of blood vessels.
(TIF)
