# Supplementary figures and images for: Oridonin Inhibits Tumor Growth and Metastasis through Anti-Angiogenesis by Blocking the Notch Signaling
Source: PLoS One. 2014 Dec 8;9(12):e113830. doi: 10.1371/journal.pone.0113830 (PMC4259472; doi:10.1371/journal.pone.0113830)

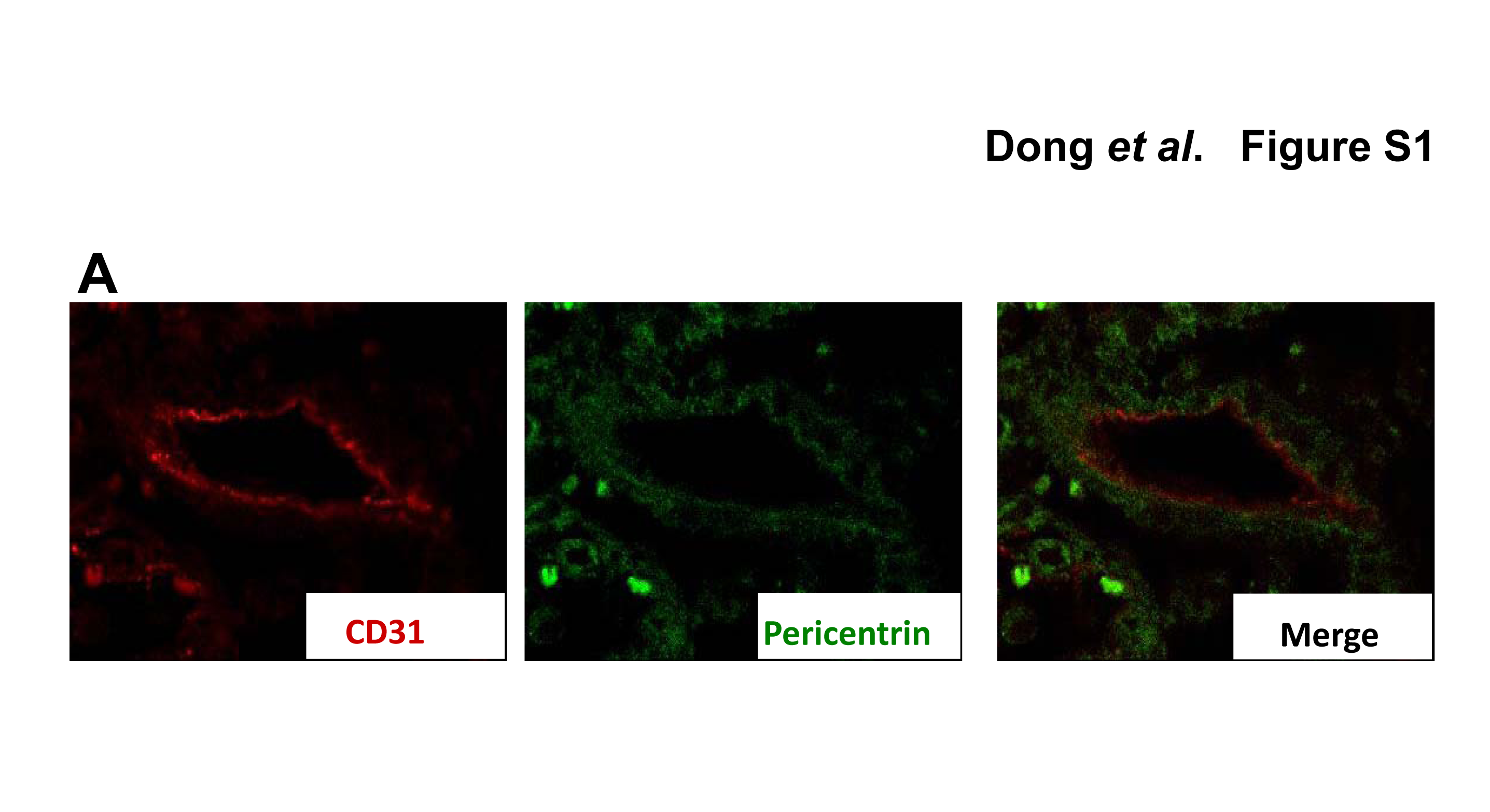

Supplement: S1 Figure — Oridonin inhibited tumor metastasis through blocking tumor cell trans-endothelium. (A) Typical images of lungs in control group. No breast cancer cells are inside of blood vessels. (TIF) [file pone.0113830.s001.tif]
